# Supplementary material for: Temperature increase drives critical slowing down of fish ecosystems
Source: PLoS One. 2021 Oct 20;16(10):e0246222. doi: 10.1371/journal.pone.0246222 (PMC8528280; doi:10.1371/journal.pone.0246222)
Supplement: S1 Table — Species ID considering the Maizuru dataset (raw data available at https://www.dropbox.com/s/q0z9hiaw9t752hx/Maizuru_dominant_sp_var.xlsx?dl=0), scientific and common name, categorization in terms of fish stock, location endemicity (native/invasive), and reported IUCN conservation status (up to December 2020). Status is provided in three categories: Not Evaluated (NE), Near Threatened (NT), Least Concern (LC). Source: http://fishbase.sinica.edu.tw/. All models, scripts for visualizations, data and results are at https://github.com/HokudaiNexusLab/FishCommunity. (PDF) [file pone.0246222.s001.pdf]

**Species**	**Fish Stock**	**Native/Invasive**	**Conservation Status**	**Other Use**	**Threat**
1. *Aurelia aurita* (Moon jellyfish)	Yes	Native	NE	Ornamental	Venomous
2. *Engraulis japonicus* (Japanese anchovy)	Yes	Native	LC	Aquaculture/Game	-
3. *Plotosus lineatus japonicus* (Sea catfish)	No	Invasive	NE	-	Venomous
4. *Sebastes inermis* (Black snapper)	Yes	Native	LC	Game	-
5. *Trachurus japonicus* (Horse mackerel)	Yes	Native	NT	Aquaculture	-
6. *Girella punctata* (Blackeye seabream)	Yes	Native	NE	Game	-
7. *Pseudolabrus sieboldi* (Wrasse)	Yes	Native	LC	-	-
8. *Halichoeres poecilopterus* (Rainbow wrasse)	Yes	Native	LC	Aquaculture/Game/Ornamental	-
9. *Halichoeres tenuispinnis* (Chinese wrasse)	No	Invasive	LC	Ornamental	-
10. *Chaenogobius gulosus* (Goby)	No	Native	NE	-	-
11. *Pterogobius zonoleucus* (Blue/Yellow striped Goby)	No	Native	LC	-	-
12. *Tridentiger trigonocephalus* (Chameleon Goby)	No	Native	NE	-	-
13. *Siganus fuscescens* (Rabbitfish)	Yes	Invasive	LC	Aquaculture	Venomous
14. *Sphyraena pinguis* (Red barracuda)	Yes	Native	NE	-	-
15. *Rudarius ercodes* (Pigmy filefish)	No	Invasive	LC	-	-

Table S1:
