# Supplementary material for: Temperature increase drives critical slowing down of fish ecosystems
Source: PLoS One. 2021 Oct 20;16(10):e0246222. doi: 10.1371/journal.pone.0246222 (PMC8528280; doi:10.1371/journal.pone.0246222)
Supplement: S2 Table — (PDF) [file pone.0246222.s002.pdf]

Species	OTE	ITE	Mean	Std
1. *Aurelia aurita*	6.477	9.612	23.826	126.050
2. *Engraulis japonicus*	8.158	3.412	68.056	379.300
3. *Plotosus lineatus japonicus*	7.058	3.174	28.253	133.030
4. *Sebastes inermis*	6.160	10.524	31.874	50.512
5. *Trachurus japonicus*	6.393	8.029	174.940	258.700
6. *Girella punctata*	6.051	7.767	14.863	29.911
7. *Pseudolabrus sieboldi*	6.273	10.308	7.333	6.970
8. *Halichoeres poecilopterus*	6.128	6.978	7.565	11.755
9. *Halichoeres tenuispinnis*	6.660	5.789	17.575	33.523
10. *Chaenogobius gulosus*	8.048	2.539	8.939	51.713
11. *Pterogobius zonoleucus*	6.230	6.839	18.542	77.554
12. *Tridentiger trigonocephalus*	6.986	11.227	31.458	43.289
13. *Siganus fuscescens*	7.071	3.768	4.646	25.866
14. *Sphyraena pinguis*	8.165	2.369	8.761	50.779
15. *Rudarius ercodes*	6.005	9.527	12.142	33.463

Table S2:
