# Supplementary material for: Temperature increase drives critical slowing down of fish ecosystems
Source: PLoS One. 2021 Oct 20;16(10):e0246222. doi: 10.1371/journal.pone.0246222 (PMC8528280; doi:10.1371/journal.pone.0246222)
Supplement: S3 Table — (PDF) [file pone.0246222.s003.pdf]

**Salience**	**All Temp.**	**≤10°C**	**10-15°C**	**15-20°C**	**20-25°C**	**≥25°C**
1	7→10 (0.87)	15→3 (0.33)	15→3,12→13 (0.87)	7→13 (0.73)	7→14 (0.93)	6→1,7→13 (0.87)
2	5→2 (0.73)	15→6 (0.27)	7→5,7→8,11→9 (0.80)	5→10 (0.67)	7→3,12→10 (0.87)	15→2 (0.80)
3	5→1,7→13,7→14 (0.67)	12→1,12→2,15→4 (0.20)	7→1,7→4,12→6,11→10 (0.73)	7→3 (0.53)	7→2,7→15 (0.80)	7→3 (0.6)
4	7→3,7→4,7→9,7→11 (0.53)	Many (0.13)	7→15 (0.67)	7→11 (0.33)	7→11 (0.67)	5→12 (0.53)
5	7→15 (0.40)	Many (0.07)	12→2,12→11 (0.40)	6→1,5→2,5→7 (0.27)	7→6 (0.53)	7→10 (0.47)

Table S3:
