# Supplementary figures and images for: Temperature increase drives critical slowing down of fish ecosystems
Source: PLoS One. 2021 Oct 20;16(10):e0246222. doi: 10.1371/journal.pone.0246222 (PMC8528280; doi:10.1371/journal.pone.0246222)

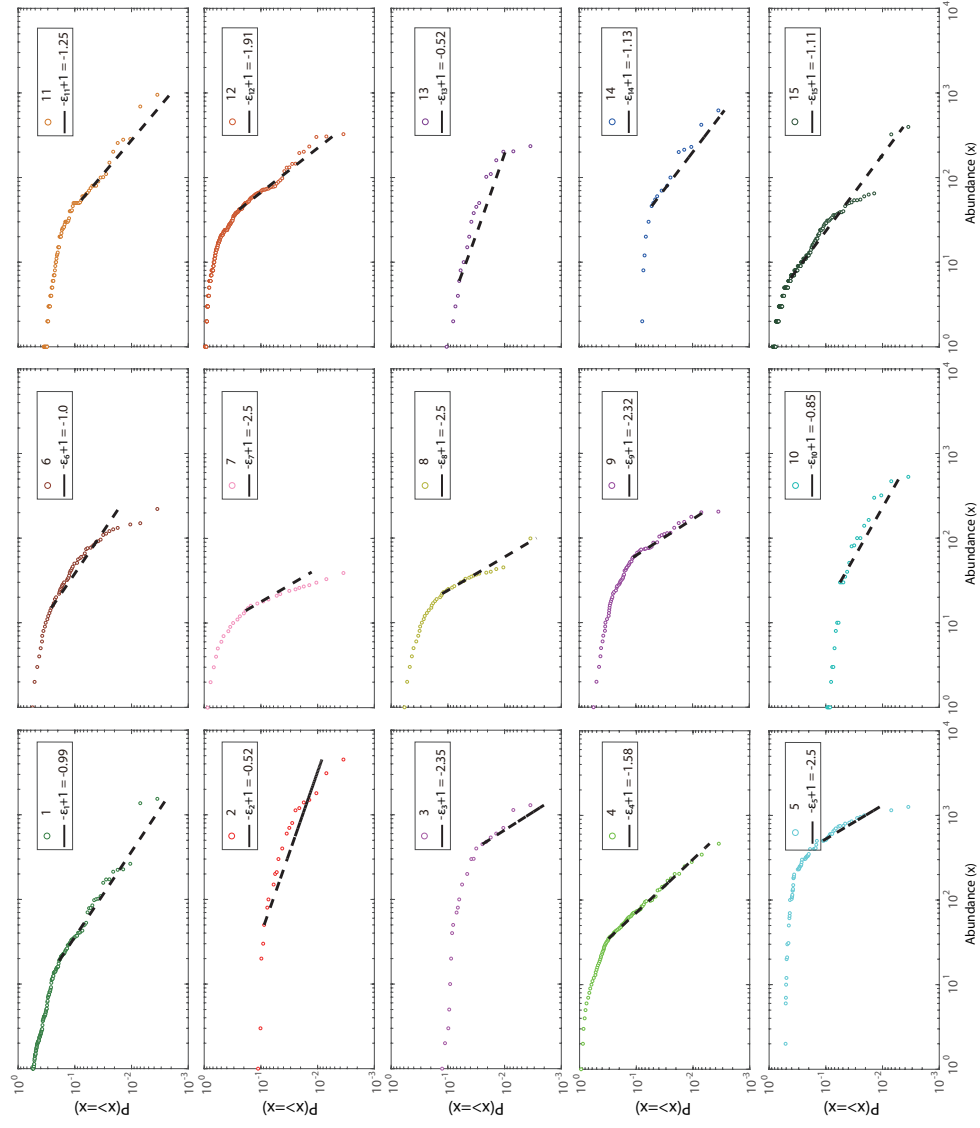

Figure S1:

Supplement: S1 Fig — Epdf of species abundance and power-law fitting. (PDF) [file pone.0246222.s004.pdf]

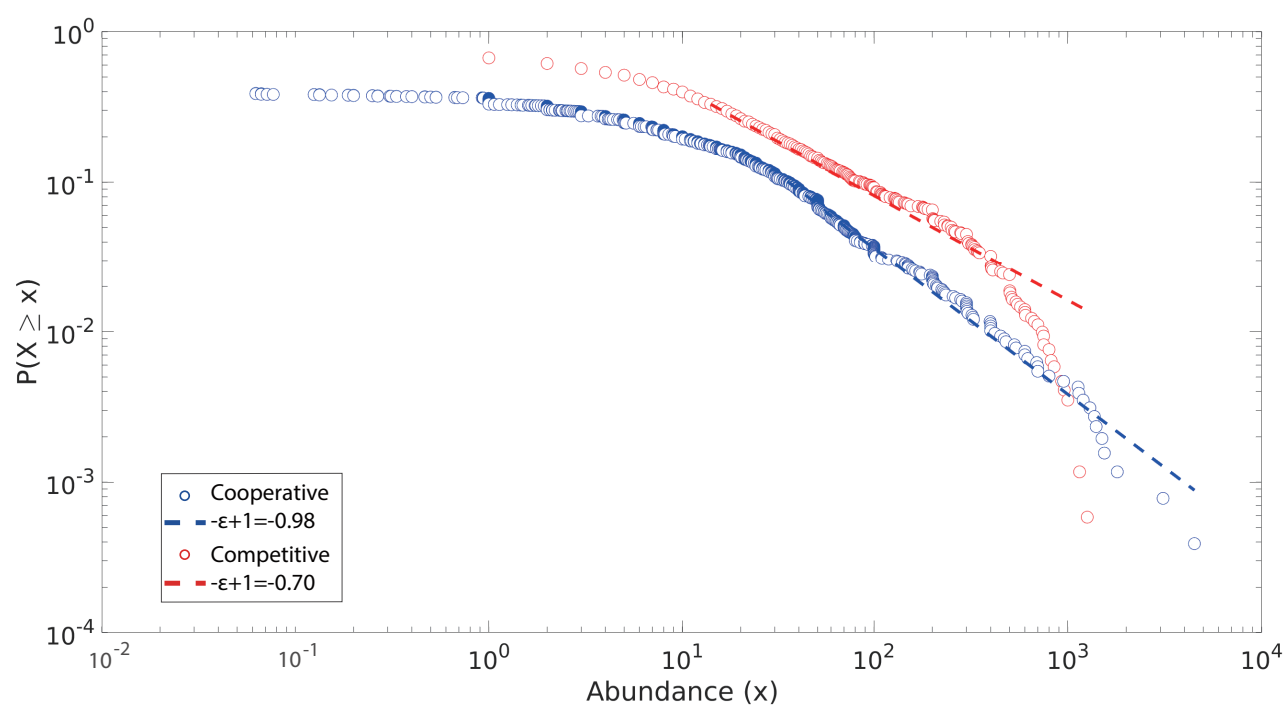

Figure S2:

Supplement: S2 Fig — Both are power-law with infinite mean and variance (critical regime) due to ϵ ≤ 2, but competitive species have a finite-size exponential decay (in the tail of the distribution) and a narrower range of criticality. This implies a more exponential set of interactions (Fig 8). Vice versa cooperative species are closer to a supercritical regime (less “heavy tail”) with larger extreme abundance and higher evenness resulting in power-law interactions. This emphasizes the duality between abundance and interactions. (PDF) [file pone.0246222.s005.pdf]

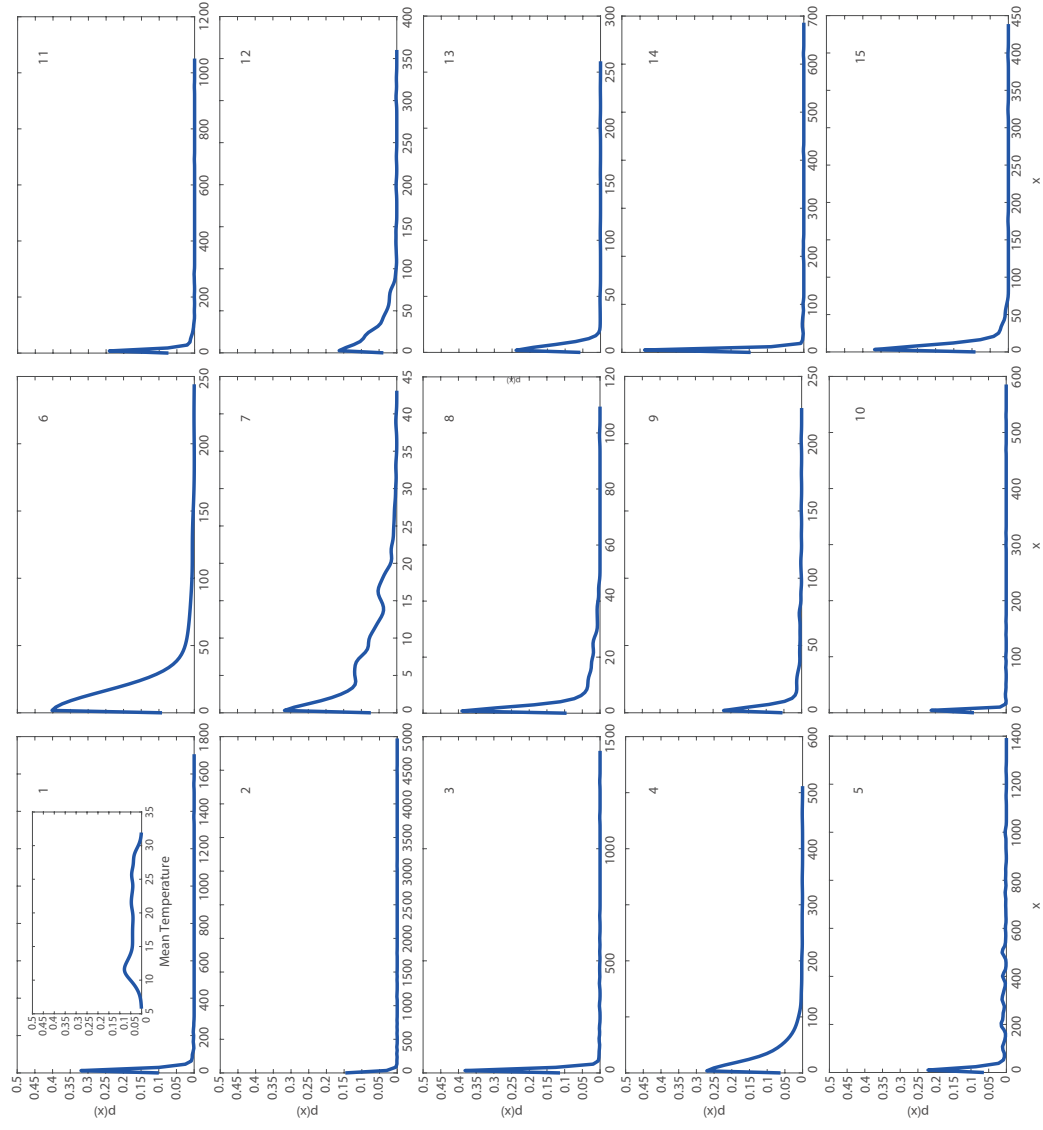

Figure S3:

Supplement: S3 Fig — The pdf of mean temperature across the whole period is shown as inset in the pdf of species 1. (PDF) [file pone.0246222.s006.pdf]

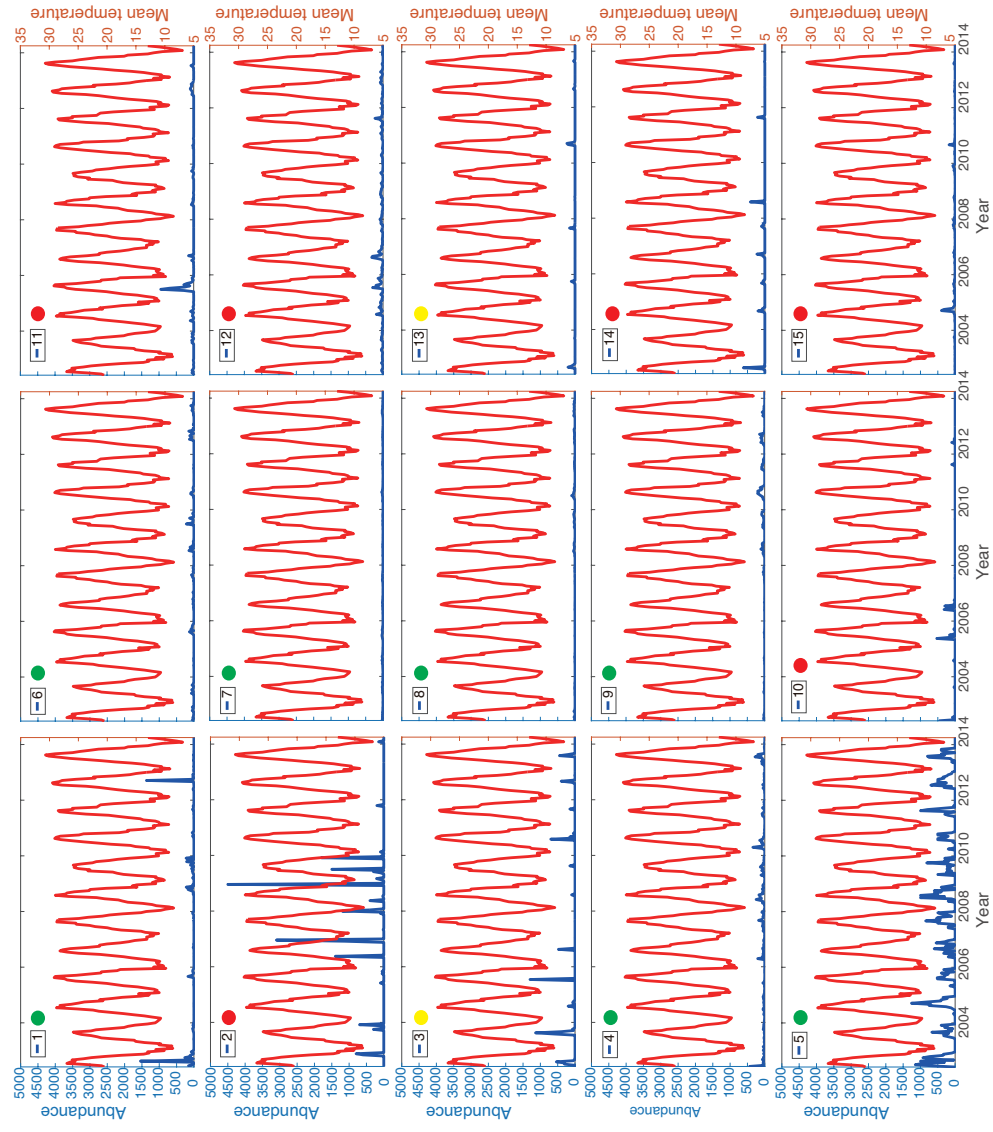

Figure S4:

Supplement: S4 Fig — Abundance is show in blue while temperate in red (equal for all species). Green, yellow, an red dots are informing about increasing, stationary, and decreasing abundance with increasing temperature considering the whole period. Note that all cooperative species (species excluding 4–9), except for species 1 (i.e. A. Aurita), are decreasing in abundance over time, that underlines their lower fitness with temperature and yet the higher impacts due to the temperature variability in conjunction with other unexplored factors. (PDF) [file pone.0246222.s007.pdf]

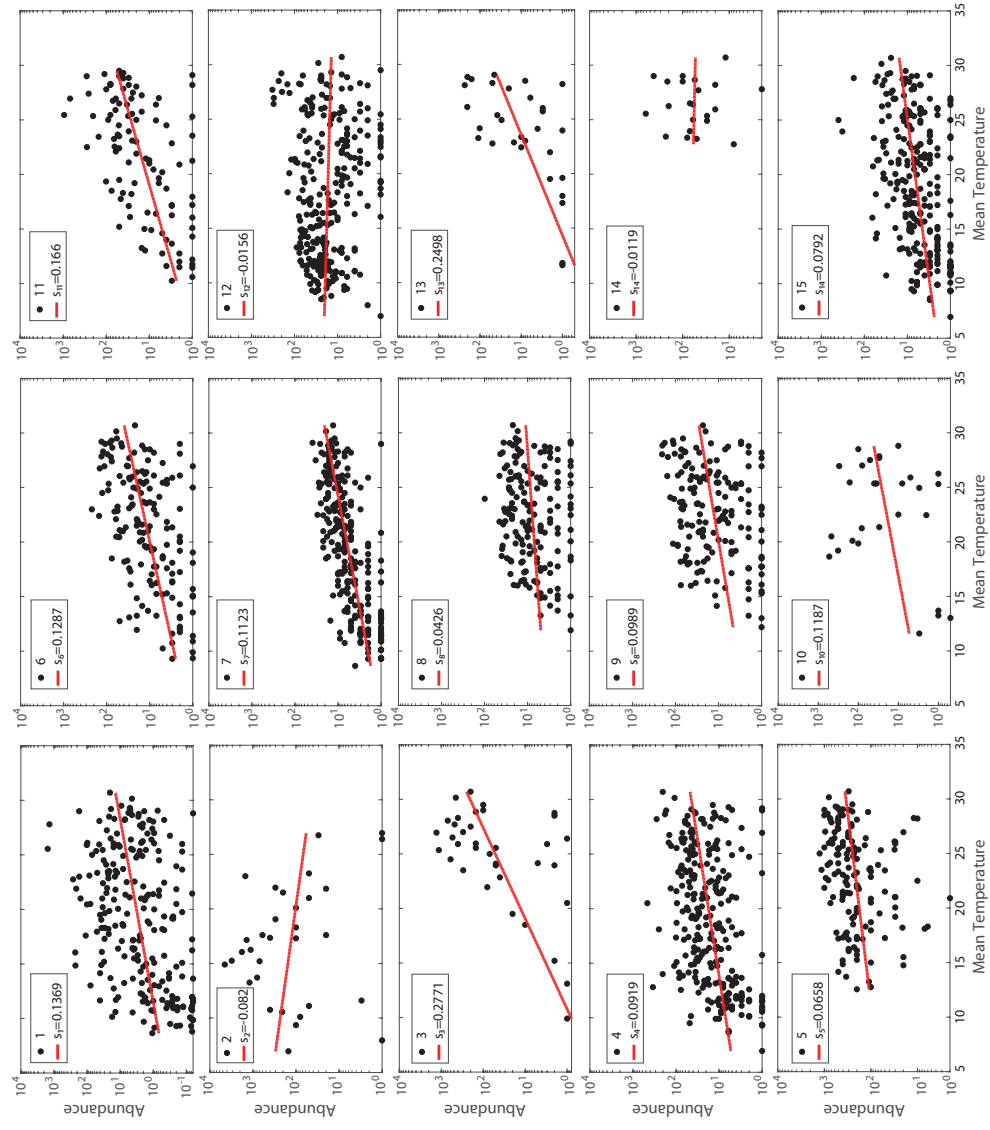

Figure S5:

Supplement: S5 Fig — Species abundance on log scale vs. mean temperature is linearly fitted a first degree polynomial model (red line). Yet, abundance is changing superlinearly with temperature causing nontrivial changes in species abundance. (PDF) [file pone.0246222.s008.pdf]

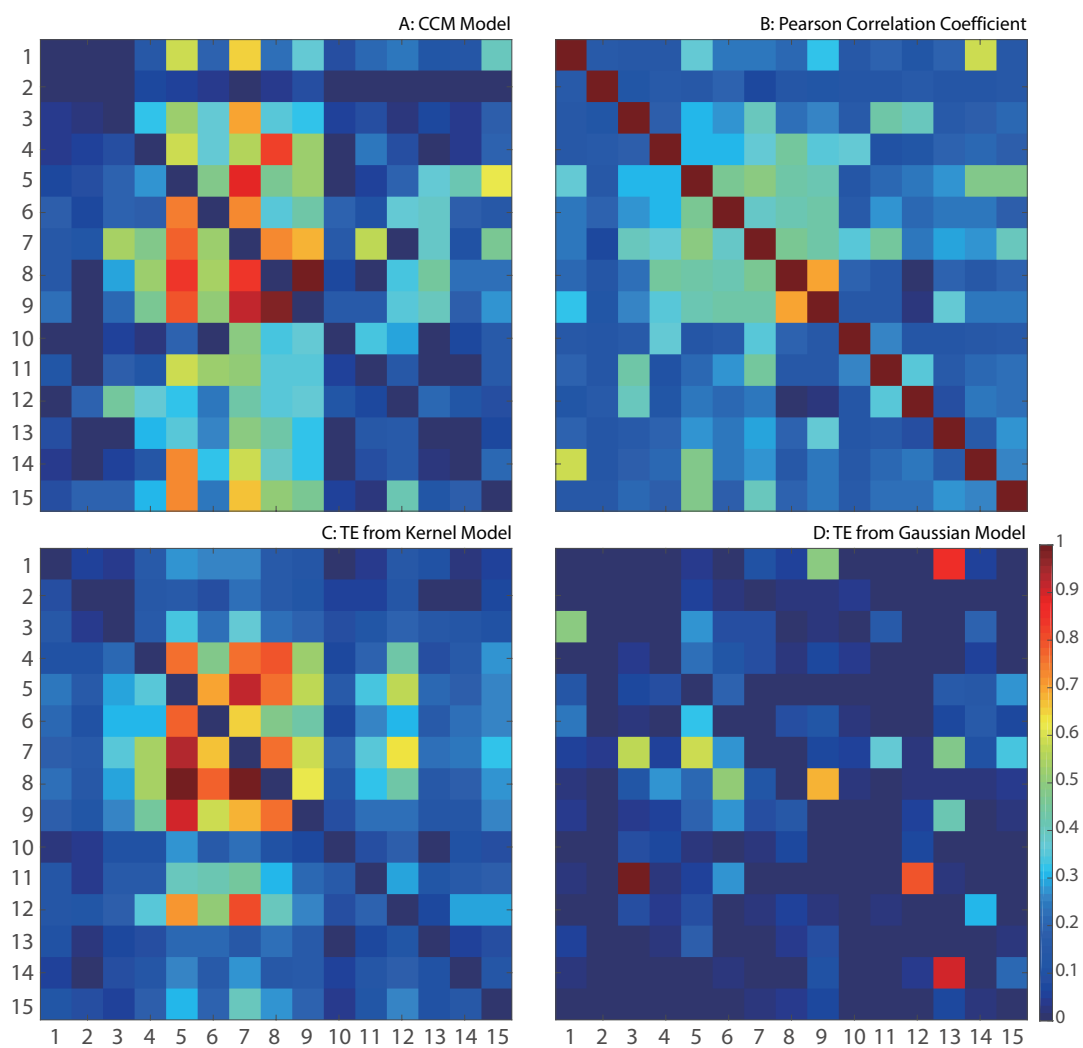

Figure S6:

Supplement: S6 Fig — A: causal interaction inference CCM model developed by [63]. B: linear association between species computed as Pearson correlation coefficient. TE-based causal interaction inference using Kernel and Gaussian estimators (C and D, respectively) in JIDT developed by [62]. (PDF) [file pone.0246222.s009.pdf]

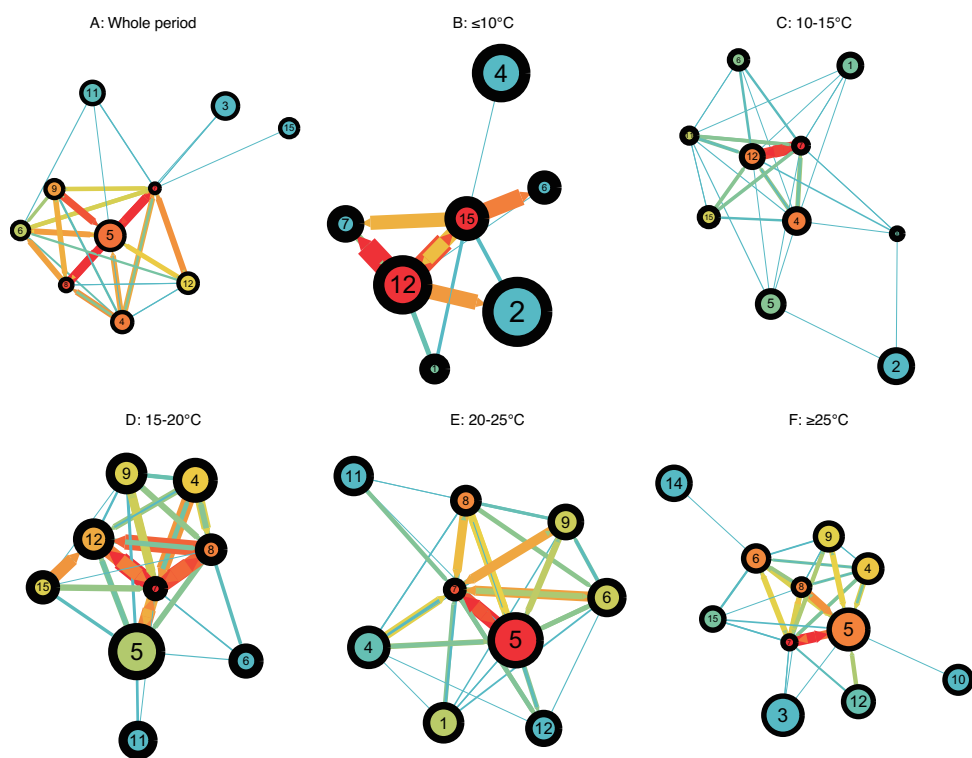

Figure S7:

Supplement: S7 Fig — The size of nodes is proportional to the Shannon Entropy of the species; the color of node is proportional to the total outgoing transfer entropies (OTE) (the higher OTE, the warmer the node’s color.); the width and color of the link between species are proportional to the TE between species pairs (the higher TE, the warmer/wider the link’s color/width). (PDF) [file pone.0246222.s010.pdf]

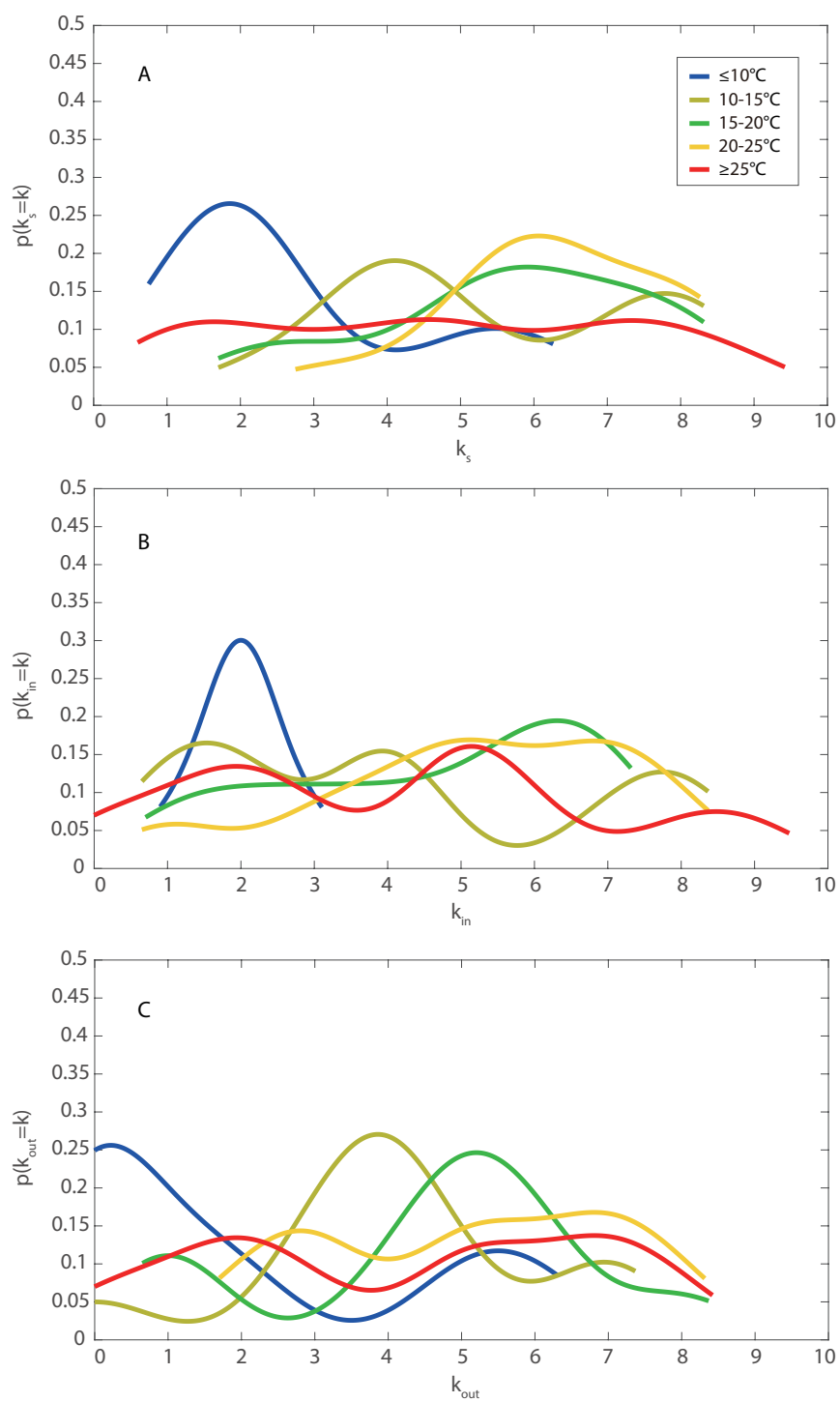

Figure S8:

Supplement: S8 Fig — A: Pdf of the structural degree, B: pdf of the in-degree, C: pdf of the out-degree, of species in OIF networks for the five TR groups. (PDF) [file pone.0246222.s011.pdf]

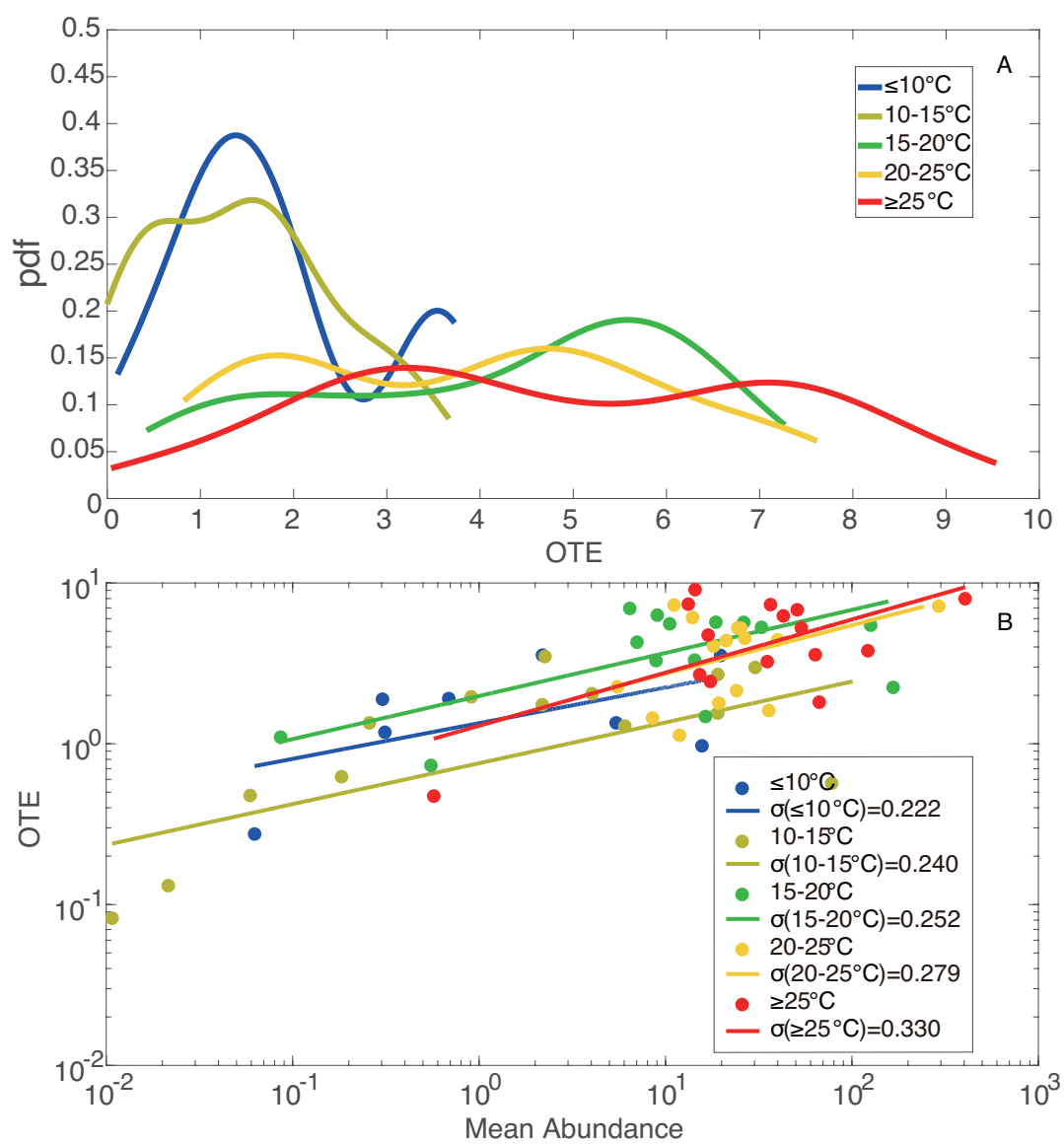

Figure S9:

Supplement: S9 Fig — A: Pdf of OTE of all species for five TR groups. B: allometric scaling of OTE as a function of mean species abundance. The latter is the community Kleiber’s law relating directed information exchange and species abundance (proportional to community metabolic rate/energy expenditure and biomass [35]). Each point refer to a species that can occur in multiple TR groups. (PDF) [file pone.0246222.s012.pdf]

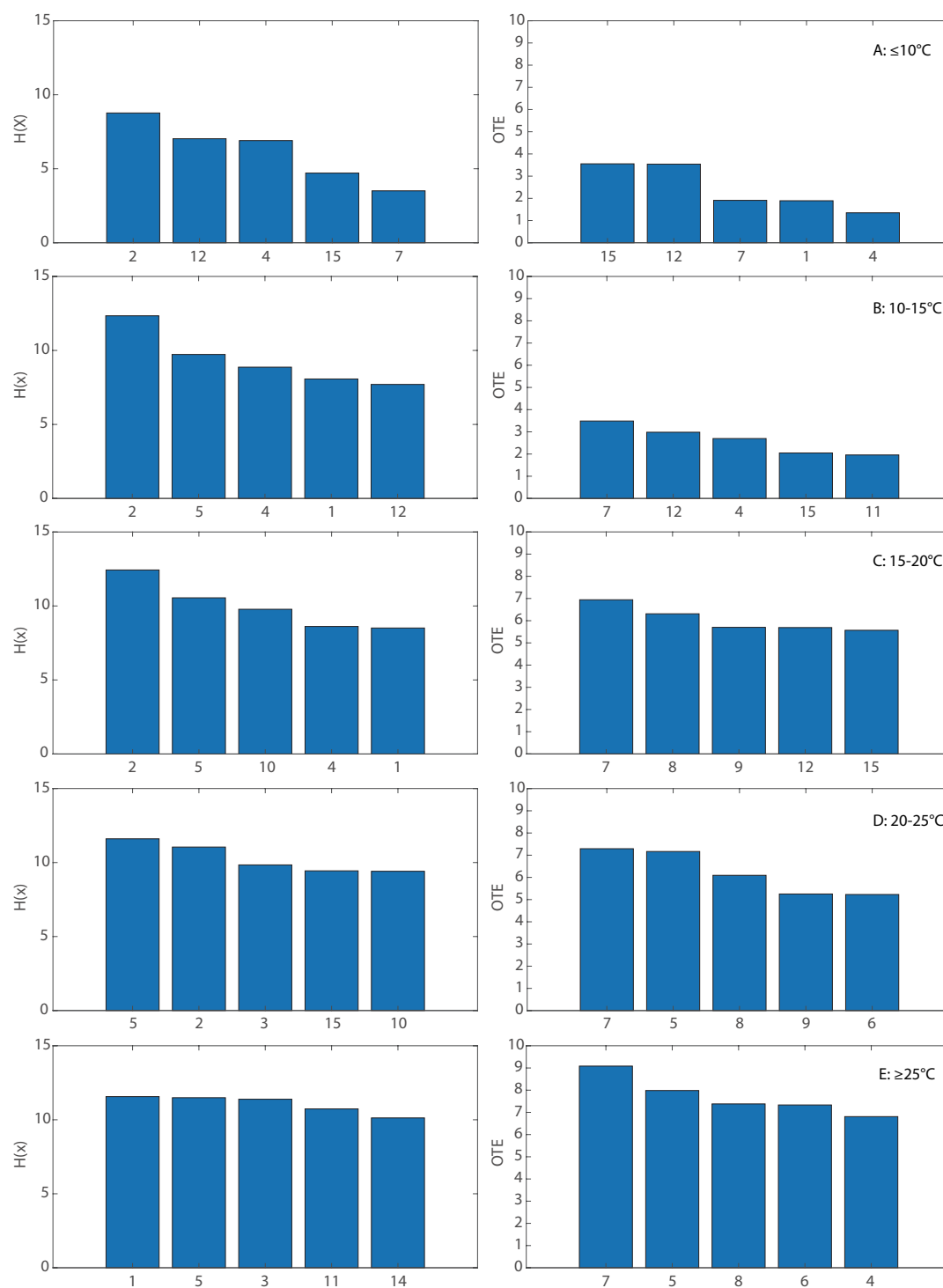

Figure S10:

Supplement: S10 Fig — Left plots show top 5 species with the highest Shannon entropy (i.e. uncertainty, or vice versa disorganized/relative information content implying lower predictability) for the five temperature ranges. Right plots show the top 5 most active species in terms of OTE (yet, affecting the community the most) for five temperature ranges. Due to the definition of salience (Eq 2.10) these are also largely coinciding with the species involved in the most salient pairs. (PDF) [file pone.0246222.s013.pdf]

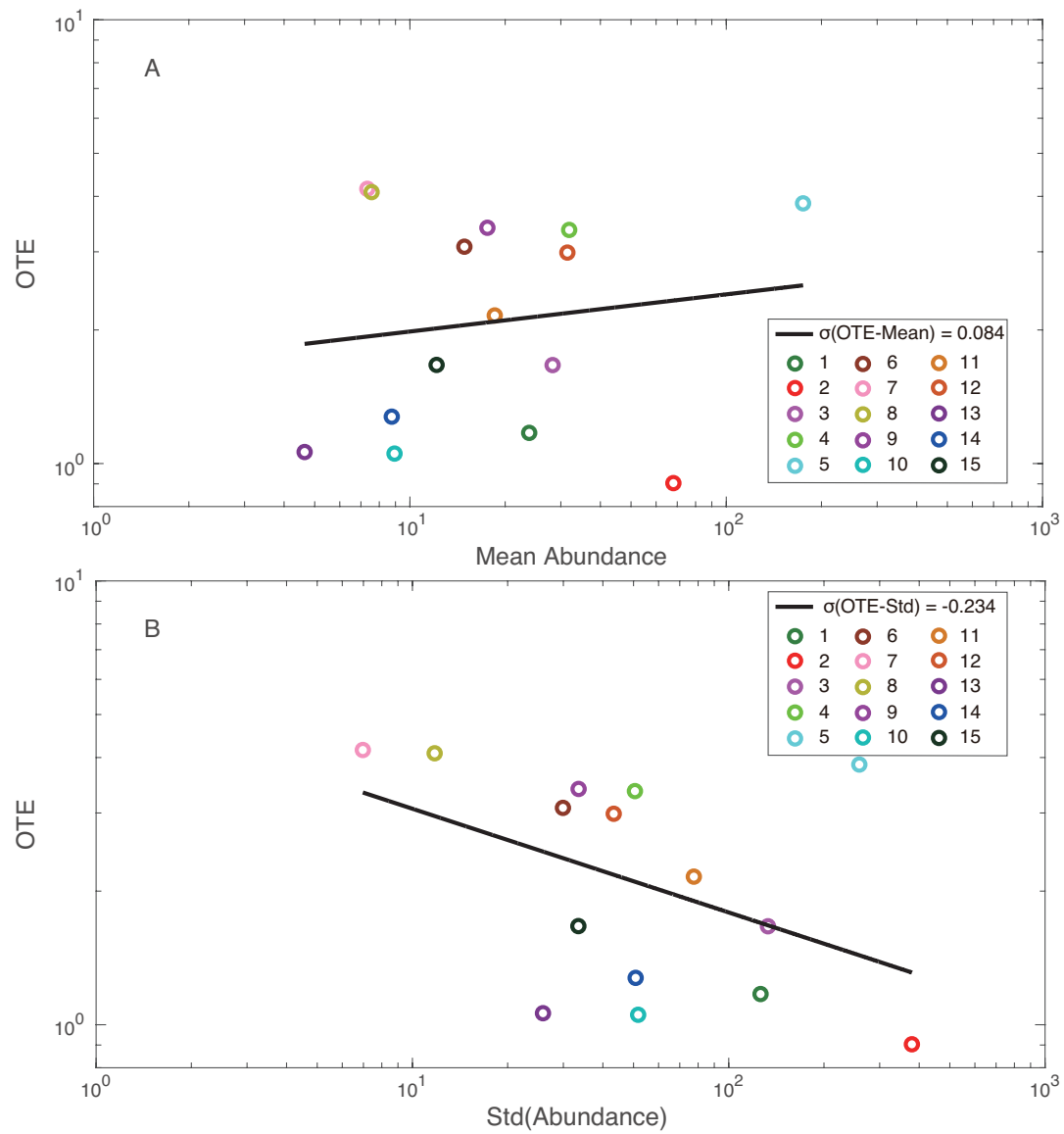

Figure S11:

Supplement: S11 Fig — A: community Kleiber’s law [35] at the species scale independent of temperature shows a non-significant trend of total directed interactions as a function of mean abundance. Vice versa, B shows how directed interactions are tendentially higher for rarer species whose fluctuations and mean of abundance are small. This supports the duality between abundance and interaction patterns and the fact that species popularity and species with extreme fluctuations (especially if asynchronous from the collective like for species 2) are not determining with Pareto interactions. In simple terms, species commonality does not increase inter-species interactions with all others but rather likely intra-species interactions only. (PDF) [file pone.0246222.s014.pdf]

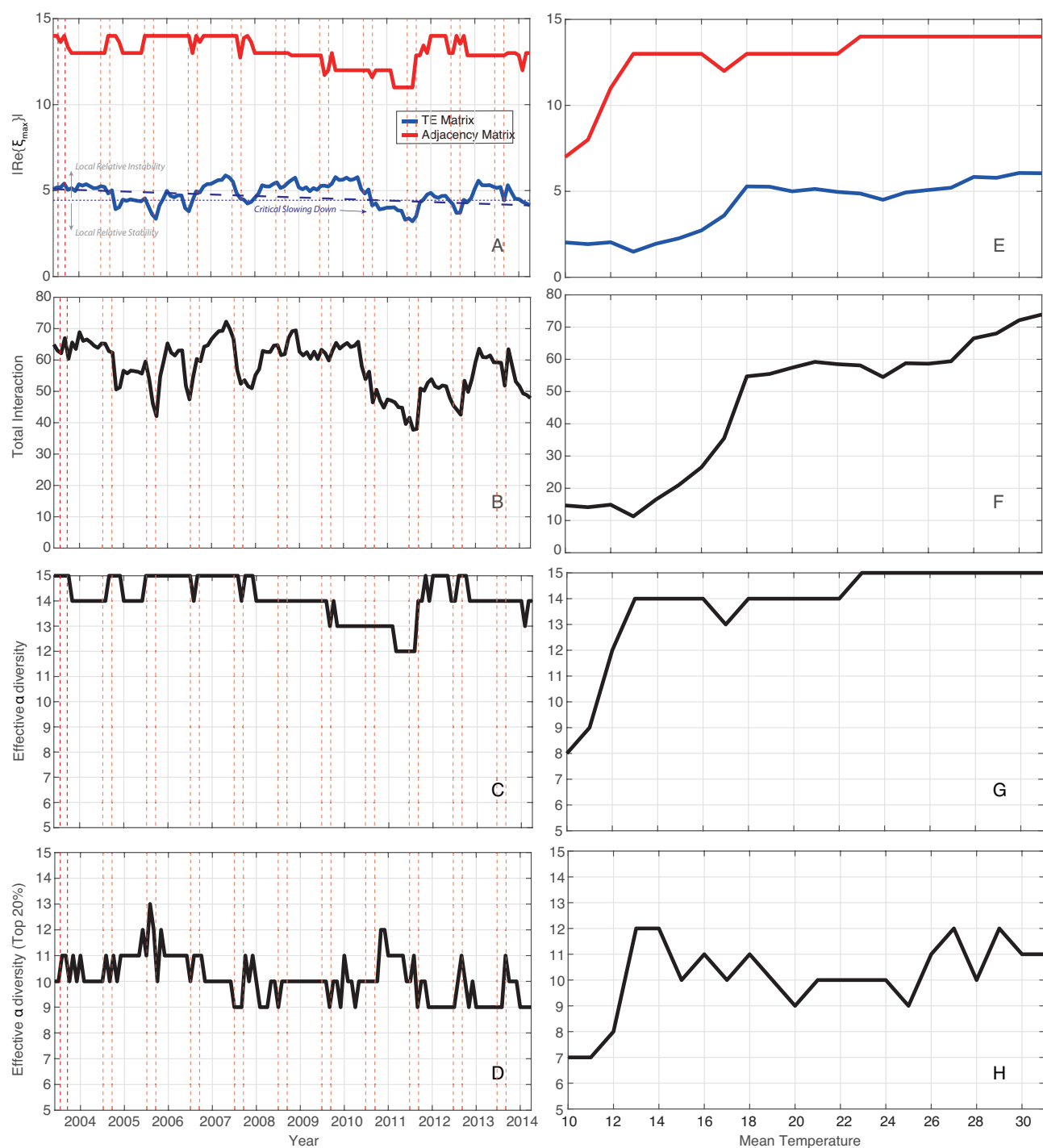

Figure S12:

Supplement: S12 Fig — The stability of the fish ecosystem is indicated by the dominant eigenvalues of the TE interaction matrix. The total interactions are calculated as the sum of TE values in the TE matrix. Effective α-diversity is the number of connected nodes (species) in dynamical networks derived from OIF-inferred TE matrices; statistically insignificant TEs define non-connected species. Dashed red lines emphasized the highest temperature/summer periods. A: Real part of the dominant eigenvalue from temporally dynamical TE matrices (blue line) and adjacency matrices (red line); B: Total interactions of temporally dynamical TE interaction matrices; C: Effective α-diversity of temporally dynamical TE interaction matrices without threshold (certain species were taxonomically non reported at certain times); D: Effective α-diversity of temporally dynamical TE interaction matrices for top 20% TEs; E: Real part of the dominant eigenvalue of temperature-dependent TE and adjacency matrices (blue and red line, respectively); F: Total interactions of temperature-dependent TE interactions; G: Effective α-diversity of temperature-dependent TE interactions without threshold; H: Effective α-diversity of temperature-dependent TE interactions for top 20% TEs. (PDF) [file pone.0246222.s015.pdf]

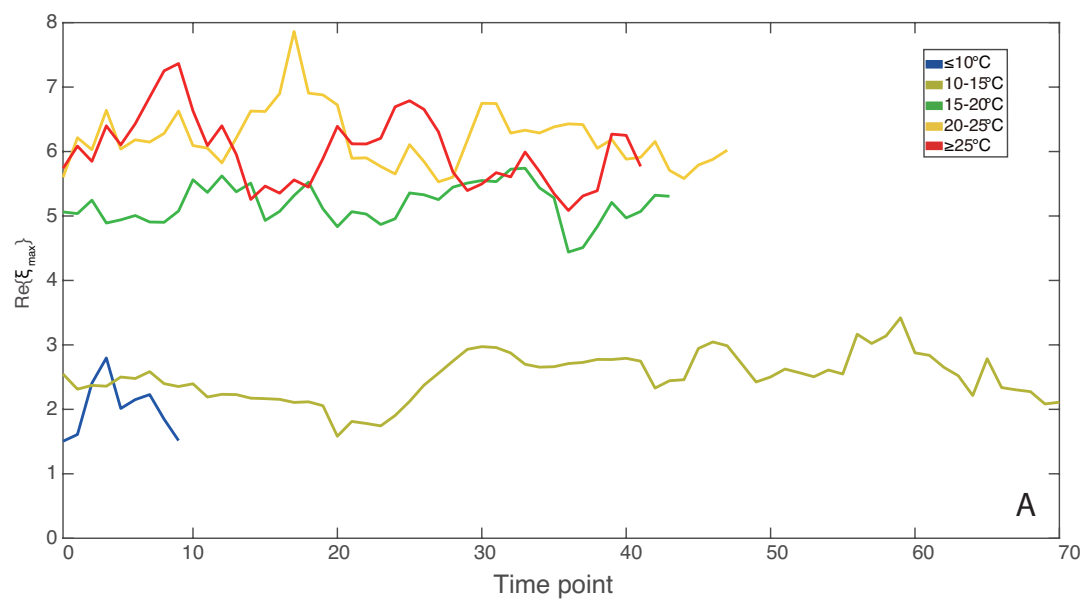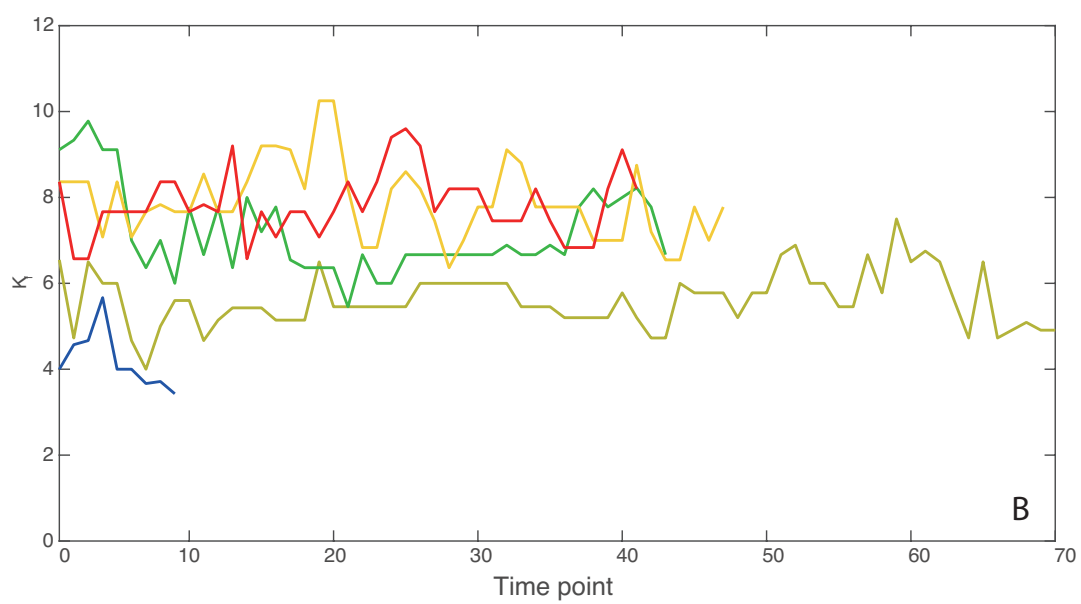

Figure S13:

Supplement: S13 Fig — The real part of the dominant eigenvalue of time-varying interaction matrices is calculated for different temperature groups. Species abundance values within each temperature groups are used independently to calculate the dominant eigenvalue. The lowest TR has the shortest time series due to the limited duration of winters in the Maizuru Bay. (PDF) [file pone.0246222.s016.pdf]

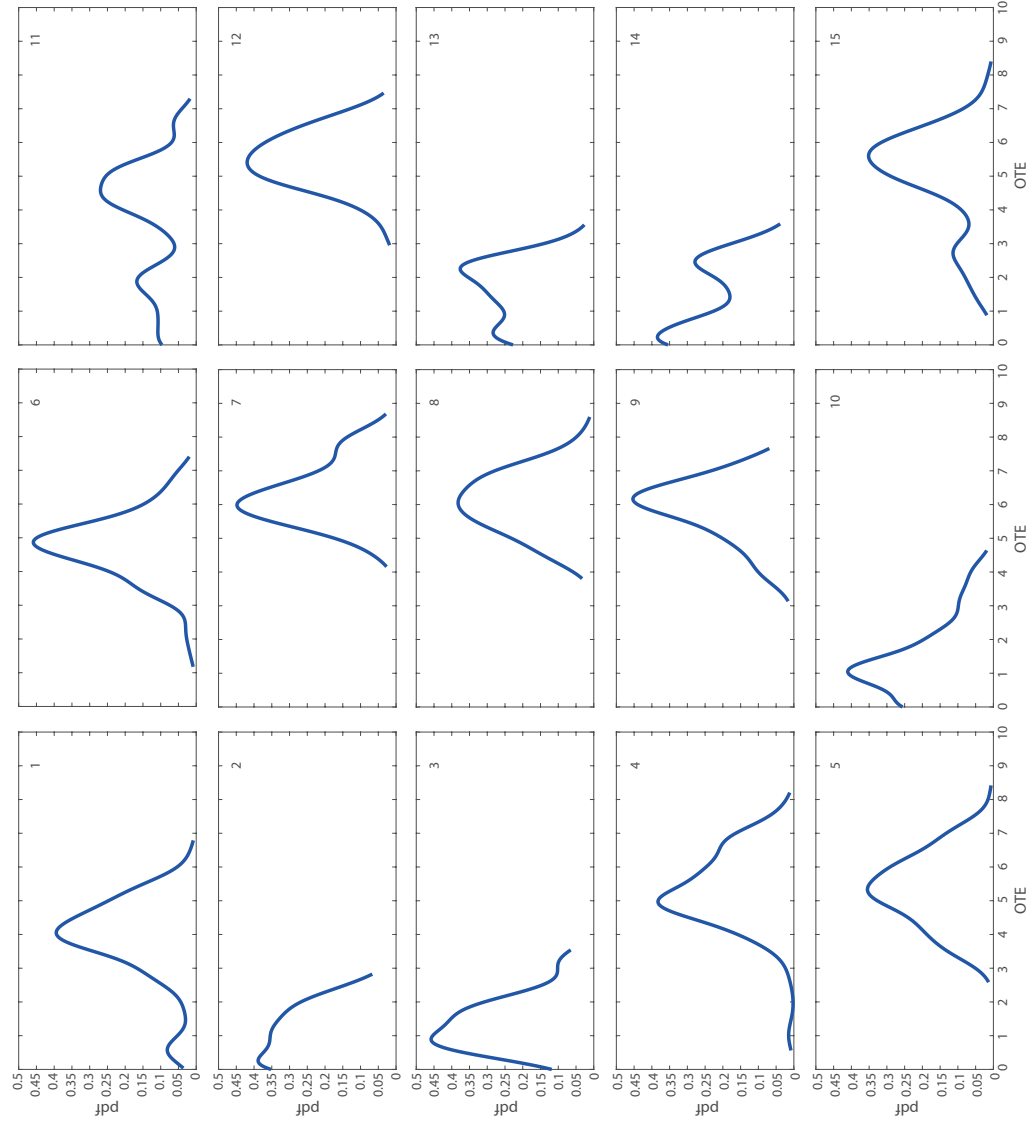

Figure S14:

Supplement: S14 Fig — Interactions are inferred via the OIF model of [28] considering the whole time series. (PDF) [file pone.0246222.s017.pdf]

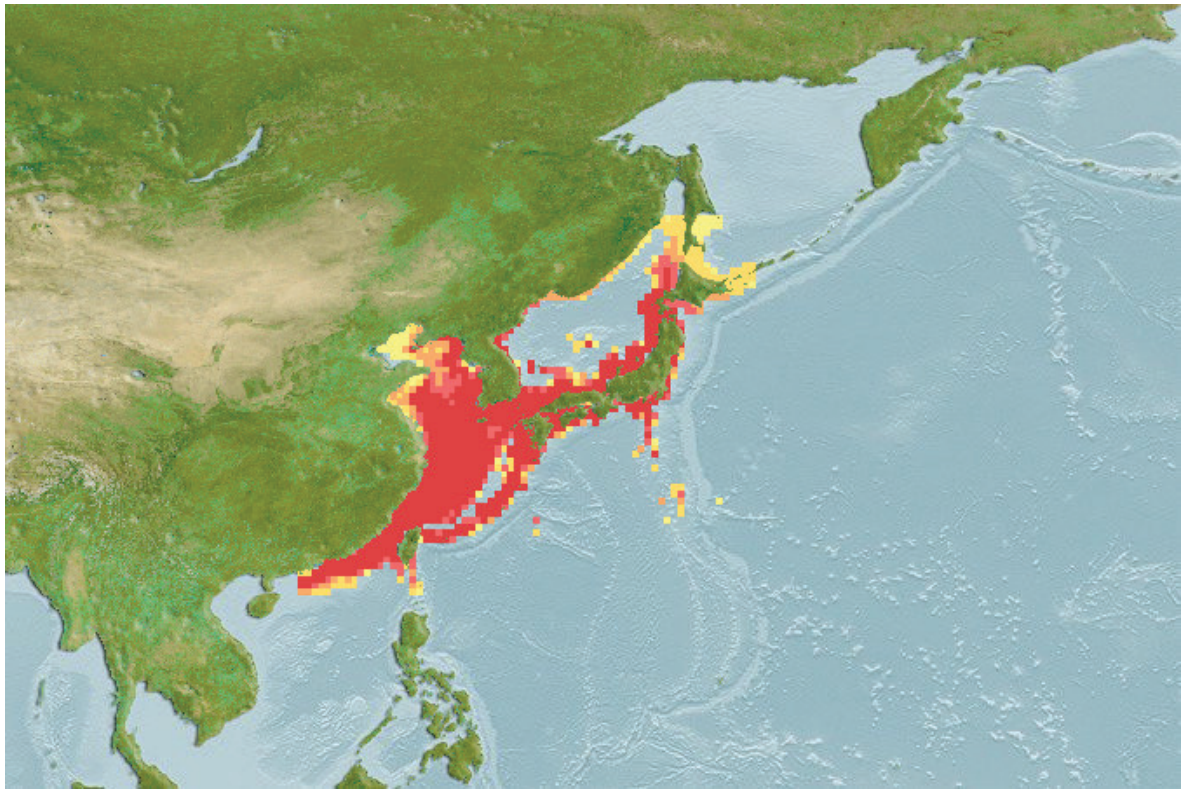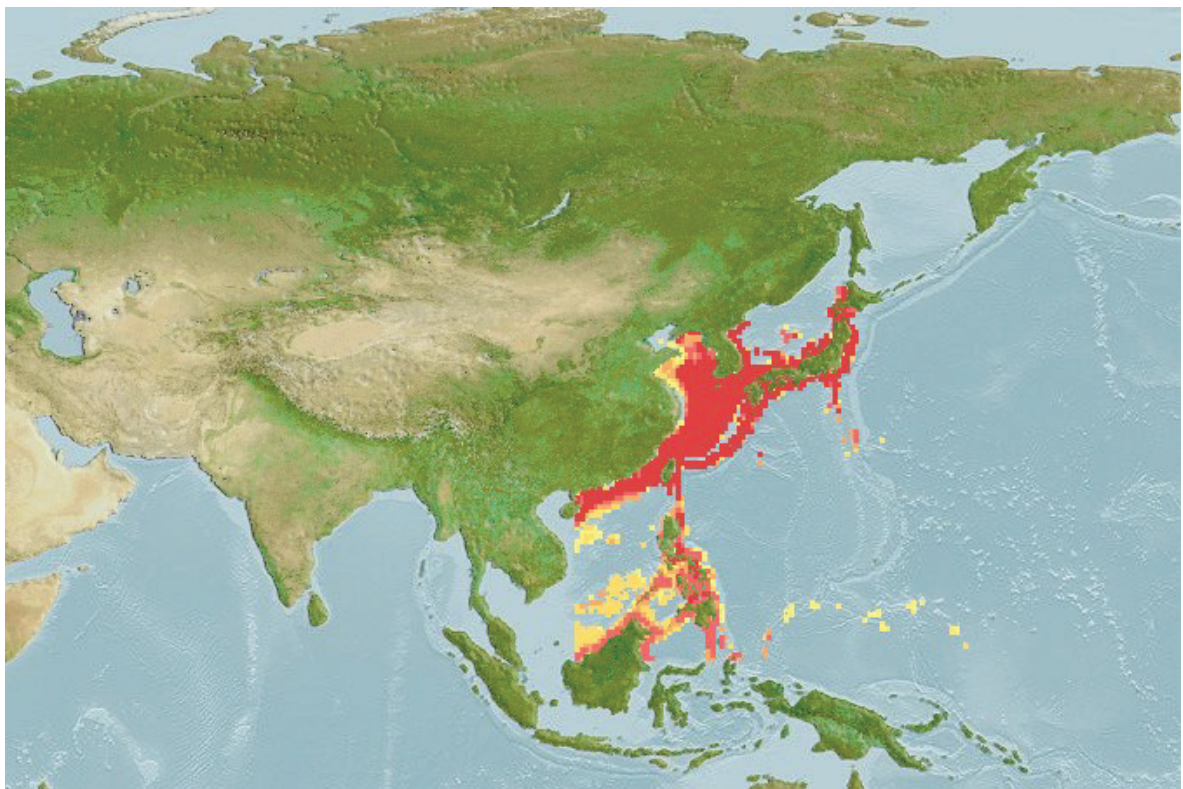

Figure S15:

Supplement: S15 Fig — Native species with cooperative behavior characterized by low directed interactions (OTE), salience (for encompassing connections), and asynchronized decreasing abundance for growing temperature. Time series are persistent, irreversible over time on average, and with low entropy. Fishing peaks (in winter) are asynchronized with temperature fluctuations but synchronized with abundance; thus, temperature is likely a second-order determinant of abundance. Abundance over time and habitat suitability (map) is power-law distributed. Source: AquaMaps; Reviewed Native Distribution Map for Engraulis japonicus (Japanese anchovy); Retrieved June 01, 2021 from http://www.aquamaps.org/preMap.php?cache=1&SpecID=Fis-29947 (material licensed under a Creative Commons Attribution-NonCommercial 3.0 Unported License). (PDF) [file pone.0246222.s018.pdf]

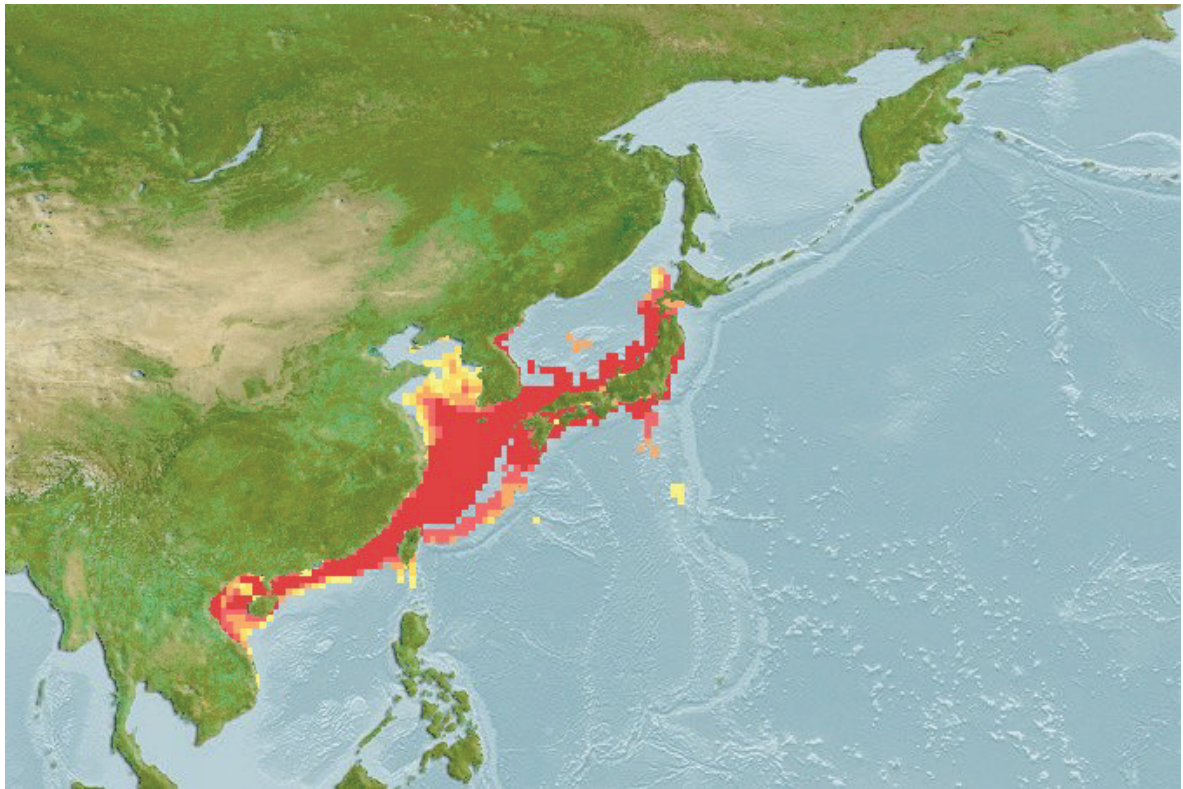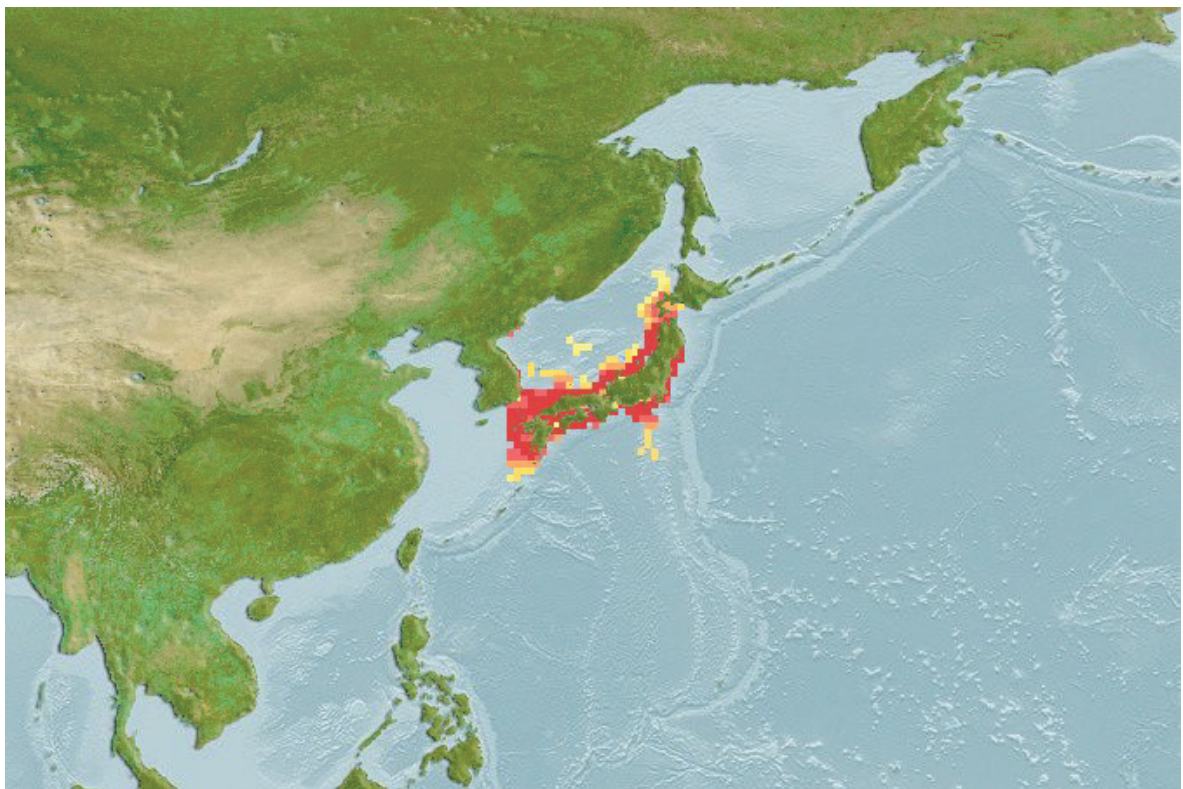

Figure S16:

Supplement: S16 Fig — Native species with competitive behavior characterized by high directed interactions (OTE), salience (for encompassing connections), and synchronized increasing abundance for growing temperature (implying lower ecological memory). Time series are antipersistent, reversible over time on average, and with high entropy. Fishing peaks (in summer) are synchronized with temperature fluctuations and abundance; thus, abundance fluctuations, driven by fishing and temperature, overlap and temperature is likely a first-order determinant. Abundance over time and habitat suitability (map) is more exponentially distributed with smaller autocorrelation than cooperative species. Higher connections and power-law like interactions define this species as a keystone species able to affect and predict ecosystem changes. Source: AquaMaps; Reviewed Native Distribution Map for Trachurus japonicus (Horse mackerel); Retrieved June 01, 2021 from https://www.aquamaps.org/receive.php?type_of_map=regular (material licensed under a Creative Commons Attribution-NonCommercial 3.0 Unported License). (PDF) [file pone.0246222.s019.pdf]

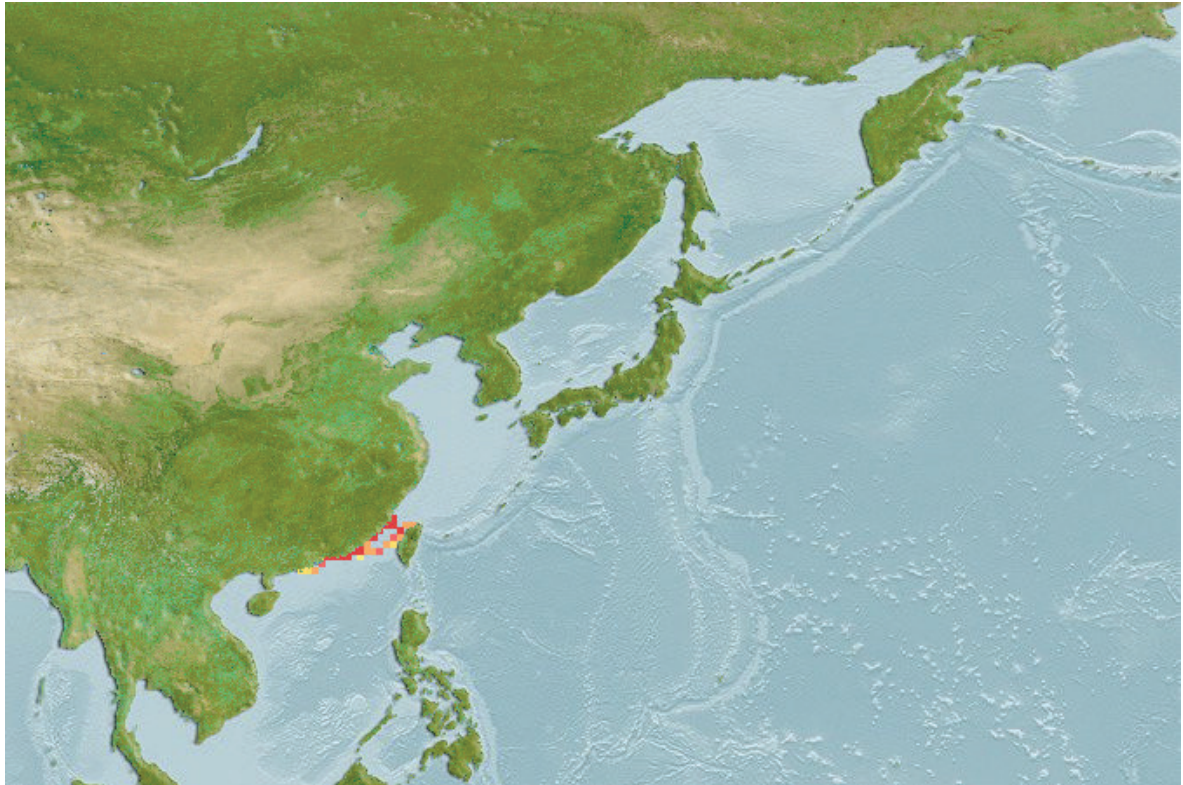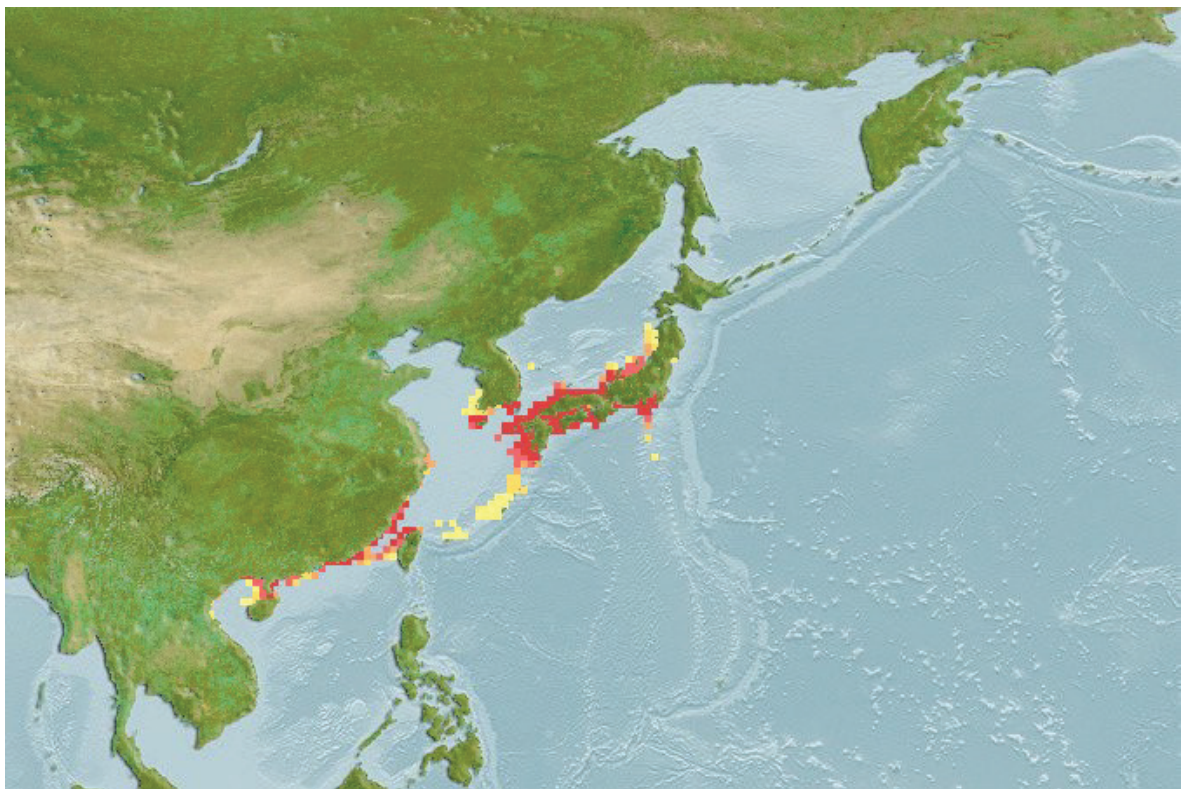

Figure S17:

Supplement: S17 Fig — Invasive species, originally from SE China, with transitory (dynamically competitive) behavior characterized by intermediate directed interactions (OTE), salience (for encompassing connections) and mildly synchronized increasing abundance for growing temperature. Abundance over time and habitat suitability (map) is exponentially distributed with fat-tail and bimodal autocorrelation emphasizing the transitory dynamics. Source: AquaMaps; Reviewed Native Distribution Map for Halichoeres tenuispinnis (Chinese wrasse); Retrieved June 01, 2021 from https://www.aquamaps.org/receive.php?type_of_map=regular (material licensed under a Creative Commons Attribution-NonCommercial 3.0 Unported License). (PDF) [file pone.0246222.s020.pdf]
