# Supplementary material for: Temperature increase drives critical slowing down of fish ecosystems
Source: PLoS One. 2021 Oct 20;16(10):e0246222. doi: 10.1371/journal.pone.0246222 (PMC8528280; doi:10.1371/journal.pone.0246222)
Supplement: S1 File — (PDF) [file pone.0246222.s021.pdf]

Temperature Increase Drives Critical Slowing Down of Fish Ecosystems
# – Supplementary Information –

Jie Li[a], Matteo Convertino[b*]

[a] Nexus Group, Laboratory of Information Communication Networks, Graduate School of Information Science and Technology, Hokkaido University, Sapporo, JP

[b] Institute of Environment and Ecology, Tsinghua Shenzhen International Graduate School (Tsinghua SIGS), Tsinghua University, Shenzhen, China

September 16, 2021

Corresponding author: * M. Convertino, Tsinghua Shenzhen International Graduate School, University Town of Shenzhen, Tsinghua Park, Nanshan District, Shenzhen 518055 P.R. China, email: matconv.uni@gmail.com

Keywords: fish community, abundance time series, OIF model, interaction networks, dominant eigenvalue, temperature, criticality, critical transitions, dynamical stability

1 Supplementary Results

As proven in previous paper (Li and Convertino, 2021) we provide a broader analysis of species interaction inference results for different TE-estimator models. Fig. S5 shows how the Gaussian estimator provides a quite different pattern of interactions than the other three models (TE with Kernel estimator, CCM and the Pearson correlation coefficient). This is likely because the Gaussian estimator is based on a linearity assumption, meaning that it assumes linear interactions between variables (Lizier, 2014). The pearson correlation coefficient is a symmetrical measure of linear correlation between two sets of data. This measure only captures linear relationship between variables, it is not capable of distinguishing directed interactions, either. Even though ρ from CCM and TE from OIF with Kernel estimator present similar patterns, the OIF-inferred heat map from Kernel presents larger gradient of interactions that highlight the divergence in fish populations of species 4-9 from other species compared to the heat map from CCM. This divergence in the distribution of species populations can be observed in Fig. S2. Therefore, OIF with Kernel estimator allows a better identification of species clusters considering gradients of inferred interactions. Additionally, TE from Kernel estimator is able to estimate some weak observed interactions such as of species 2 with others, while CCM essentially consider null interactions for these species. Therefore, the Kernel estimator was selected as the TE estimator in the OIF model (Li and Convertino, 2021).

References

Jie Li and Matteo Convertino. Inferring ecosystem networks as information flows. Scientific Reports, 11(1):1–22, 2021.

Joseph T. Lizier. Jidt: An information-theoretic toolkit for studying the dynamics of complex systems. Frontiers in Robotics and AI, 1:11, 2014. ISSN 2296-9144. doi: 10.3389/frobt.2014.00011. URL https://www.frontiersin.org/article/10.3389/frobt.2014.00011.
